# Mechanism for direct graphite-to-diamond phase transition

**DOI:** 10.1038/srep05930

**Published:** 2014-08-04

**Authors:** Hongxian Xie, Fuxing Yin, Tao Yu, Jian-Tao Wang, Chunyong Liang

**Affiliations:** 1 School of Mechanical Engineering, Hebei University of Technology, Tianjin 300132, China; 2 Research Institute for Energy Equipment Materials, Hebei University of Technology, Tianjin 300132, China; 3 Central Iron and Steel Research Institute, Beijing 100081, China; 4 Beijing National Laboratory for Condensed Matter Physics, Institute of Physics, Chinese Academy of Science, Beijing 100190, China

## Abstract

Using classical molecular dynamics with a more reliable reactive LCBOPII potential, we have performed a detailed study on the direct graphite-to-diamond phase transition. Our results reveal a new so-called “wave-like buckling and slipping” mechanism, which controls the transformation from hexagonal graphite to cubic diamond. Based on this mechanism, we have explained how polycrystalline cubic diamond is converted from hexagonal graphite, and demonstrated that the initial interlayer distance of compressed hexagonal graphite play a key role to determine the grain size of cubic diamond. These results can broaden our understanding of the high pressure graphite-to-diamond phase transition.

As the hardest material in nature, diamond is valuable for industrial uses, in addition to its well-known use as gemstone, which motivates the long-standing study of man-made diamonds^1^,^2^,^3^,^4^,^5^,^6^. However, forming diamond is far from easy. About ten years ago, polycrystalline diamond was successfully synthesized by direct conversion sintering from high-purity graphite under static ultrahigh pressure (above 12 GPa) and high temperature (above 2000°C) using the Kawai-type multi-anvil apparatus^7^. These works suggested that polycrystalline diamond has a mixed texture of a homogeneous fine structure (particle size: 10–20 nm) and a lamellar structure (particle size: 100–200 nm)^7^,^8^,^9^,^10^,^11^. It has also been explained that diamond particles in the homogeneous fine structure are transformed from graphite in a diffusion process, while diamond layers in the lamellar structure are transformed in a martensitic process from graphite^8^,^9^. However, further experiments revealed that the homogeneous fine structure is also transformed in a martensitic process instead of diffusion process^12^.

Despite the fact that the mechanism for direct graphite-to-diamond phase transition has been the subject of intense theoretical research^13^,^14^,^15^,^16^,^17^,^18^,^19^,^20^,^21^, the underlying microscopic mechanisms of how polycrystalline diamond is converted from high-purity graphite has remained enigmatic and called for further theoretical exploration. Molecular dynamics simulation is considered to be a powerful tool for solving these puzzles. However, because of the high computational cost associated with *ab initio* or tight-binding-based molecular dynamics simulations, the simulation system is too small (less than a few hundreds atoms), this will lead to the artificial sliding of graphite layers in small-cell simulations^16^,^22^. On the other hand, in the past classical molecular dynamics simulation was hindered due to the inability of empirical potentials to describe the graphite phase accurately. Fortunately, with the improvement of empirical potentials, classical molecular dynamics simulation has become more and more powerful. Recently, using classical molecular dynamics with a more reliable NN potential nucleation mechanism for the direct graphite-to-diamond phase transition was studied^23^. The results of the simulation demonstrated that the large lattice distortions that accompany the formation of diamond nuclei inhibit the phase transition at low pressure, and direct it towards the hexagonal diamond phase at higher pressure. Obviously, the proposed nucleation mechanism can improve our understanding of structural transformations from graphite to diamond phase. However, in their works, rhombohedral graphite (RG) lattice was used as initial structures for the formation of cubic diamond nuclei. It is well known that graphite mainly exists in hexagonal rather than rhombohedral phase in nature, this brings up the question of how the hexagonal graphite transforms into cubic diamond. Beside of the NN potential, the long-range carbon bond order potential (LCBOPII) is another popular potential for carbon^24^, which not only accounts for covalent bonds in the various hybridization forms, but for weaker nonbonded interactions such as those between graphite layers, and is therefore particularly well-suited for graphite. LCBOPII potential has been applied to calculate grain boundary energy and elastic properties of graphene^25^,^26^, phonon dispersions and high-pressure high-temperature equation of state of graphite^27^,^28^; all these calculated results are in good agreement with ab initio calculations or experimental results. Another important feature of the LCBOPII potential for the present study is its ability to describe the energy barrier of the graphite-diamond transformation accurately^29^, which gives confidence in the correct description of the kinetics of direct graphite-to-diamond phase transition^30^.

In this paper, we use classical molecular dynamics^31^ with LCBOP II potential^24^ to investigate the underlying microscopic mechanism for the direct hexagonal graphite-to-diamond phase transition. The crystallographic orientations of the initial hexagonal graphite lattice in the *x-, y-* and *z*-axis are taken to be in the directions of [210], [100] and [001], respectively. First, the initial interlayer distance of hexagonal graphite lattice is set to a certain value; then the configuration is equilibrated using molecular dynamics in the isobaric-isothermal (NPT) ensemble to relax the pressure of *x* and *y* directions to 0 bar while the interlayer distance of hexagonal graphite lattice hold constant. Finally the configuration is under uniaxial compression along the *x* direction with a constant speed in the canonical (NVT) ensemble(see Methods).

We find that the compressed hexagonal graphite can directly convert to single or polycrystalline cubic diamond via a new *wave-like buckling and slipping* mechanism and the initial interlayer distance of compressed hexagonal graphite plays a key role to determine the grain size of polycrystalline cubic diamond. Moreover, our simulations show that hexagonal diamond also can be achieved from hexagonal graphite by a simple slip model, but no *wave-like buckling* occur during the compression process.

## Result

**Figure 1:**
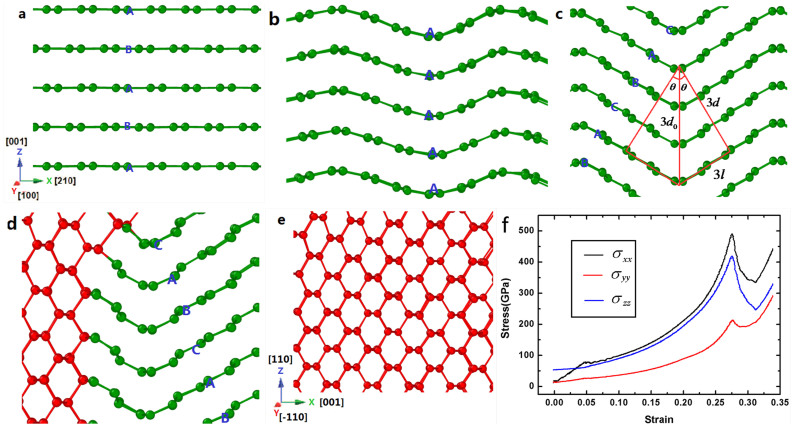
Transformation of hexagonal graphite with 0.240 nm interlayer distance into momocrystal cubic diamond. In the figure, the green and red balls represent atoms that have three and four nearest neighbors, respectively. (a). The initial state of hexagonal graphite with 0.240 nm interlayer distance. (b). The wave-like buckling of the graphite layers leads to the stacking order of the graphite layers transforming from “ABAB” to “AAAA”. (c). When the inclination angle reaches 30°, the stacking order of the graphite transforming from “AAAA” to “ABCA” (rhombohedral graphite), and the interlayer distance (*d*) can be calculated as 0.208 nm. (d). Parts of graphite convert into cubic diamond. (e). Graphite completely transforms into single-crystal cubic diamond. (f). The stress-strain curves in the compression process.

We first report the results of the simulation from compressed hexagonal graphite (with 0.240 nm interlayer distance) to perfect cubic diamond (see Fig. 1). We set pressure along the *x*[210] direction. At the early stages of structural transformation under compression, the graphite is under elastic compression, in which, the graphite layers hold straight and no slipping occur (Fig. 1a). When the compressive stress along the *x*[210] direction reaches a critical value, a “wave-like buckling” of the graphite layers occur: parts of the graphite rotate clockwise and the remaining parts rotate anticlockwise; at the same time, the slipping of the graphite layers leads to the stacking order of the graphite layers transforming from “ABAB” to “AAAA” (Fig. 1b). With the increasing of the compressive stress, the inclination angle (*θ*) of the graphite layers becomes larger and larger, meanwhile the bond length between the C-C atoms becomes shorter and shorter [the interlayer distance of the buckled graphite (*d*) can be calculated as: *d* = *d*_0_ cos*θ* (where *d*_0_ = 0.240 nm is the initial interlayer distance of the hexagonal graphite); and the ‘slipping' distance between the adjacent graphite layers (*l*) is calculated as: *l* = *d*_0_ sin*θ*]. When the inclination angle reaches 30°, the bond length reduces to 0.128 nm; at the same time the interlayer distance of the buckled graphites can be estimated to be 0.208 nm, which is approximately equal to the {111} interplanar distance of cubic diamond (0.206 nm). The ‘slipping' distance between the adjacent graphite layers can be calculated as 0.12 nm, this leads to the stacking order of the graphite layers transforming from “AAA” to “ABC” (Fig. 1c). Then the graphite in ABC stacking begins to transform into a perfect cubic diamond through the same transformation pathways proposed in references^13^,^16^ (Fig. 1d). The graphite layers rotating clockwise and anticlockwise transform into (-11-1) and (1-1-1) planes, respectively. Consequently, the [210], [100] and [001] axes of graphite transform into the [001], [−110] and [110] directions of cubic diamond (Fig. 1e). The transformation process in detail is displayed in movie S1 (see supplementary materials). These results suggest a new “wave-like buckling and slipping” transformation mechanism, which is distinct from the usual zigzag and armchair buckling conversion mechanism for the cold-compressed graphite phase transformation proposed by Wang et al.^32^.

The stresses are also monitored during the compression process. The stress-strain curves along the three directions are displayed in Fig. 1f. At the initial state, the stresses along the *x[210]* and *y[100]* direction are zero, while the stress along the *z[001]* direction is 53.3 GPa (because the interlayer distance of the graphite has been compressed to 0.240 nm). At the elastic compression stage, the stress along the *x* direction increases linearly with increasing strain up to critical wave-like buckling stress, which is larger than the stress along the *z* direction. Then the stresses of the three directions all increase with the increasing of strain; finally the stress along the *x* direction reaches its maximum (488.7 GPa) and then drops suddenly; at the same time the graphite begins to transform into cubic diamond.

**Figure 2:**
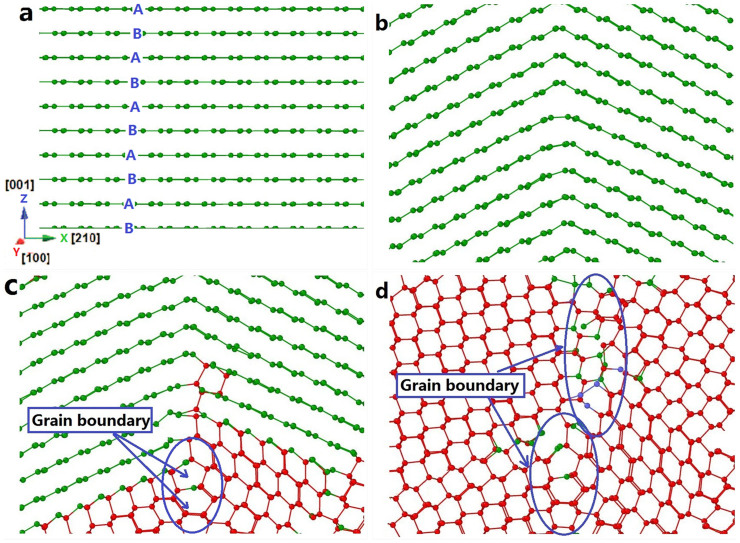
Transformation of hexagonal graphite with 0.214 nm interlayer distance into polycrystalline cubic diamond. In the figure, the green and red balls represent atoms that have three and four nearest neighbors, respectively. (a). The initial state of hexagonal graphite with 0.214 nm interlayer distance. (b). The wave-like buckling of the graphite layers leads to the stacking order of the graphite layers transforming from “ABAB” to “ABCA” (rhombohedral graphite). (c). Parts of graphite convert into cubic diamond. (d). Graphite completely transforms into polycrystalline cubic diamond.

To understand the transformation mechanism from graphite to polycrystalline diamond, the structural transformation process with interlayer distance of 0.214 nm are also shown in Fig. 2. We can see that the hexagonal graphite layers first buckle at a critical compressive stress, and in the meantime the slipping of the graphite layers leads to the stacking order of the graphite layers transforming from “ABAB” to “AAAA”. With the increasing of the compressive stress, the inclination angle of the graphite layers becomes larger and larger; when the inclination angle reaches a certain value of 23.5°, the stacking order of the graphite layers transforming from “AAAA” to “ABCA” (Fig. 2b). Then, the rhombohedral graphite begins to transform into polycrystal cubic diamond; the atoms near the buckling place form grain boundary (Fig. 2c) with local displacement. As a result, hexagonal graphite transforms into polycrystal cubic diamond (Fig. 2d) with buckling angle smaller than 30°.

**Figure 3:**
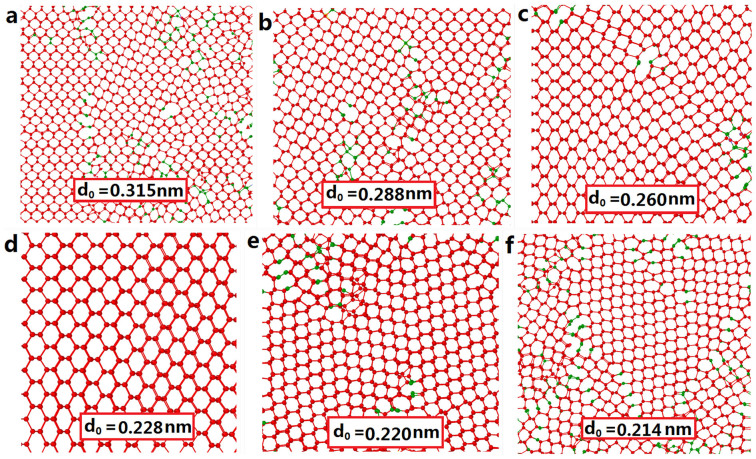
The obtained finial structure of cubic diamond after compression of hexagonal graphite with different interlayer distances. In the figure, the green and red balls represent atoms that have three and four nearest neighbors, respectively.

The phase transformation from hexagonal graphite to diamond with different interlayer distances of 0.320 nm–0.208 nm has been studied. Our simulations show that when the interlayer distance of the hexagonal graphite is between 0.240–0.228 nm, the graphite will transform into a perfect crystal of cubic diamond with a critical buckling angle of 30°. However, when the interlayer distance of the hexagonal graphite is larger than 0.240 nm or less than 0.228 nm with the buckling angle of the graphite layers larger or smaller than 30°, the graphite will transform into polycrystalline cubic diamond; Moreover, when the interlayer distance of the hexagonal graphite is less than 0.208 nm, it will be converted into hexagonal diamond rather than cubic diamond. We here illustrate the final configurations in Fig. 3 with interlayer distance of 0.315 nm, 0.288 nm, 0.260 nm, 0.228 nm, 0.220 nm and 0.214 nm. The more detailed transformation patterns can be found in movies S2–S7 (see supplementary materials).

**Figure 4:**
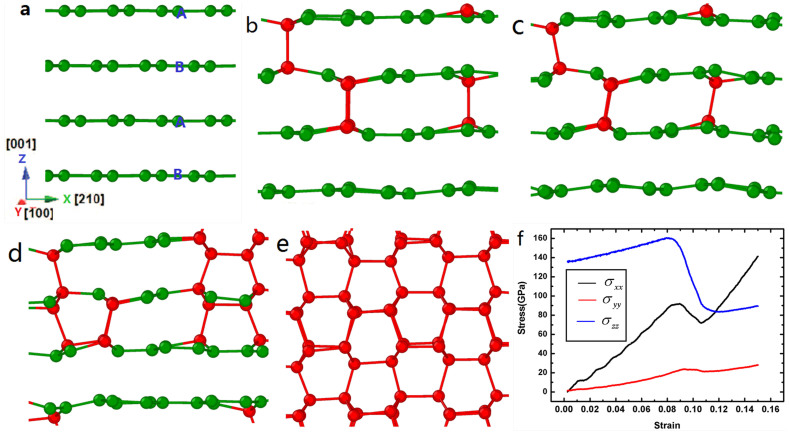
Transformation of hexagonal graphite with 0.208 nm interlayer distance into hexagonal diamond, in the figure the green and red balls represent atoms that have three and four nearest neighbors, respectively. (a). The initial state of hexagonal graphite. (b). The configuration of hexagonal graphite after 10 ps relaxation. (c). Hexagonal graphite has slipped into orthorhombic graphite phase. (d). Parts of graphite convert into cubic diamond. (e). Graphite completely transforms into single-crystal hexagonal diamond. (f). The stress-strain curves in the compression process.

To get the best understanding, the conversion process from hexagonal graphite to hexagonal diamond with 0.208 nm interlayer distance is also given in Fig. 4. At the initial stage after 10 ps relaxation 18% atoms of the hexagonal graphite (Fig. 4b) have four nearest neighbors. With the increasing of compressive stress, the hexagonal graphite layers gradually slip into intermediate orthorhombic graphite phase, then the orthorhombic graphite further transforms into hexagonal diamond phase through the same transformation pathways proposed in references^13^,^16^. It is noted that the wave-like buckling of the graphite layers does not occur during this compression process. The transformation process in detail is shown in movie S8 (see supplementary materials). The stress-strain curves of the three directions during the compression process are displayed in Fig. 4f. At the initial state, the stresses along the *x* and *y* directions are zero, and the stress along the *z* direction is 136.2 GPa. During the compression process, the stress along the *x* direction increases linearly with increasing strain. When the stress along the *x* direction reaches 92.1 GPa, the graphite begins to transform into hexagonal diamond. These results show a strong z[001]-axis compression process in contrary to cubic diamond phase conversion shown in Fig. 1f.

**Figure 5:**
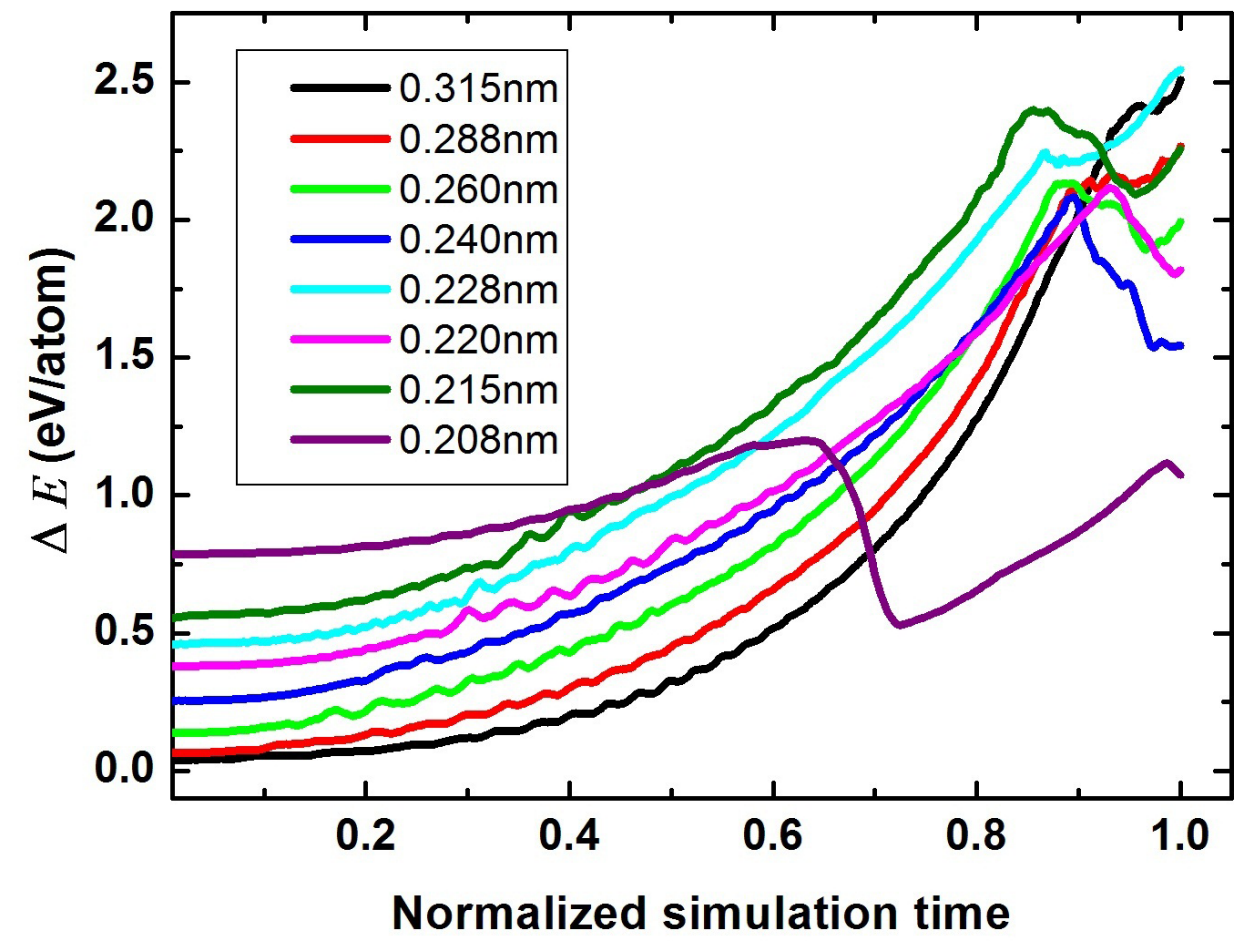
The change of potential energy versus the normalized simulation time during the compression process.

The change of per atom potential energy during the transformation process can be calculated following the equation 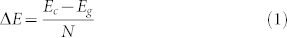
where *E_c_* is the current potential energy of the sample, *E_g_* is the initial state potential energy of the sample (the hexagonal graphite at 0 bar), *N* is the total atom number of the sample. Fig. 5 gives the change of per atom potential energies versus the normalized simulation time during the eight compression processes. From the figure it can be found that the maximum energies for interlayer distance from 0.288 nm to 0.220 nm are about 2.11 eV, which is about 0.20 eV lower than that for interlayer distance 0.315 nm and 0.215 nm (2.41 eV); this can be attributed to different distortion of graphite to be made for different interlayer distance. It also can be see that the maximum energy for interlayer distance 0.208 nm (1.18 eV) is much lower than those for other interlayer distances, which indicates that it is much easier to form hexagonal diamond than to form cubic diamond from compressing hexagonal graphite, this is because the high symmetry of the hexagonal graphite to hexagonal diamond transformation pathway.

## Discussion

In summary, a new “wave-like buckling and slipping” mechanism is proposed to probe into the atomistic mechanism for the direct graphite-to-cubic diamond phase transition. Based on the “wave-like buckling and slipping” mechanism, we have explained how polycrystalline cubic diamond can be converted from hexagonal graphite. We found that the initial interlayer distance of the compressed hexagonal graphite determines the grain size of cubic diamond. Our simulation work provides a possible pathway for the high pressure graphite-to-diamond phase transition.

## Methods

The MD simulations are performed using the LAMMPS code^31^ with LCBOP II potential^24^. Simulations are carried out by integrating Newton's equations of motion for all atoms using a time step of 0.5 fs. The crystallographic orientations of the initial hexagonal graphite lattice in the *x-, y-* and *z*-axis are taken to be in the directions of [210], [100] and [001], respectively (the initial length of *x* direction is 13.2 nm, while the *y* and *z* directions' length are 10.2 nm and 13.6 nm, respectively, containing 192,000 atoms). The initial interlayer distance of hexagonal graphite lattice is set to a certain value; then the configuration is equilibrated using molecular dynamics in the isobaric-isothermal (NPT) ensemble at certain temperature (2000 K) for 10 ps; during the thermally equilibrium, the interlayer distance of hexagonal graphite lattice hold constant and relaxed the pressure of *x* and *y* directions to 0 bar (LAMMPS code has the ability to hold specific dimension not changed in the isobaric-isothermal (NPT) ensemble). Finally the configuration was compressed along the *x* direction with a constant speed (100 *m*/*s*) in the canonical (NVT) ensemble (fix indent command of the LAMMPS code is used to realize the compression process). During the compression process, the periodic boundary conditions were used in *y* and *z* directions; and the temperature of the system was 2,000 K. In this paper, engineering strain is defined as *ε* = (*l* −*l*_0_)/*l*_0_, where *l* is the current length and *l*_0_ is the length along the *X* direction after the thermally equilibrium process. The stresses reported in this work were calculated using the virial theorem, which takes the form: 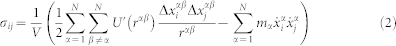
Where V is the current volume of the sample, N is the total number of atoms, 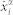 is the *i*th component of velocity of atom *α*, *m_α_* is the mass of atom *α*, *r^αβ^* is the distance between two atoms *α* and *β*, 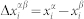, *U* is the potential energy function.

## Author Contributions

H.X., F.Y., T.Y. and J.T.W. performed the simulation and wrote the main manuscript text. H.X. and C.L. prepared the figures and videos. All authors reviewed the manuscript. The authors have no competing financial interests.

## Supplementary Material

Supplementary Information S1

Supplementary Information S2

Supplementary Information S3

Supplementary Information S4

Supplementary Information S5

Supplementary Information S6

Supplementary Information S7

Supplementary Information S8

Supplementary Information supplementary video legends
