# Supplementary material for: Mechanism for direct graphite-to-diamond phase transition
Source: Sci Rep. 2014 Aug 4;4:5930. doi: 10.1038/srep05930 (PMC4120013; doi:10.1038/srep05930)
Supplement: Supplementary Information — supplementary video legends [file srep05930-s9.doc]

**Mechanism for direct graphite-to-diamond phase transition**

Hongxian Xie^1,2,α)^, Fuxing Yin^2^, Tao Yu^3^, Jian-Tao Wang^4,β)^ , Chunyong Liang^2^

1. *School of Mechanical Engineering, Hebei University of Technology, Tianjin 300132, China*
2. *Research Institute for Energy Equipment Materials, Hebei University of Technology, Tianjin 300132, China*
3. *Central Iron and Steel Research Institute, Beijing 100081, China*
4. *Beijing National Laboratory for Condensed Matter Physics, Institute of Physics, Chinese Academy of Science, Beijing 100190, China*

  The supplementary videos named S1-0.240nm, S2-0.315nm, S3-0.288nm, S4-0.260nm, S5-0.228nm, S6-0.220nm, S7-0.214nm and S8-0.208nm display the compression process of hexagonal graphite with initial interlayer distances 0.240nm, 0.315nm, 0.288nm, 0.260nm, 0.228nm, 0.220nm, 0.214nm and 0.208nm, respectively.
